# Principles and Pragmatics for Building Trust in Authority: Comment on "Placing Trust at the Heart of Health Policy and Systems"

**DOI:** 10.34172/ijhpm.8964

**Published:** 2025-04-23

**Authors:** Stephen David Reicher

**Affiliations:** School of Psychology and Neuroscience, University of St. Andrews, St. Andrews, UK.

**Keywords:** Trust, Community, Social identity, Social Influence, COVID-19

## Abstract

McKee et al make a powerful plea for placing trust-building at the core of public health initiatives. I endorse that call and propose one general principle along with four practical guidelines for building trust between the public and the authorities. The general principle is that trust is rooted in shared identity and that, therefore, the task of building trust is rooted in building a sense of shared identity both amongst the public and between the public and authorities. The four guidelines are (1) trust the people!; (2) recognise and respect difference; (3) engage with the public; and (4) understanding and support trump blame and punishment. Details and justifications for each of these guidelines is provided in Supplementary file 1.

## Lessons From the Pandemic

 COVID-19 provided a dramatic reminder that behaviour matters for public health. Before vaccines were developed, we could only limit the spread of infection by changing behaviour: getting people to increase their *spatial* distance from others.^1^

 When vaccines became available, it did not mean that behaviour no longer mattered. Rather, it introduced new behavioural issues, notably, whether people would get vaccinated and how to persuade them to do so. It became ever clearer that the various disciplines contributing to the COVID-19 response needed to come together in joint structures and develop a genuine dialogue: medical and life scientists addressing what behaviours would enable or inhibit infection; behavioural scientists addressing how to impact those behaviours.

 COVID-19 also illustrated the importance of getting the behavioural analysis right (both by researchers and by policy-makers). Early in 2020, for instance, the UK Government assumed that individuals would lack the resilience to put up with COVID-19 restrictions for very long. Accordingly, they delayed the introduction of stay-at-home measures.^2^ A modelling study (based on estimates of the impact of “lockdown” on infection spread) suggested that, had action been taken a week earlier, then some 30 000 lives would have been saved in the first wave of the pandemic. Had action been taken two weeks earlier (at the same time as Italy), that figure would have been 40 000.^3^

 In the event, this assumption of individual frailty was proved wrong. Levels of adherence started and remained high, even to the most stringent of measures, such as staying at home and not receiving visitors and even when adherence caused people severe difficulties^4^. When people failed to adhere it was less to do with psychological weakness than such factors as lack of confidence in Government^5^ and the lack of practical resources to make it possible.^6^

 To give just one example of the importance of trust consider the aforementioned topic of vaccination. On the one hand, vaccine uptake was strongly associated with levels of trust in governmental and health authorities.^7^ On the other hand, a tendency to explain low take-up in terms of personal failings (such as “selfishness”^8^) and hence to impose coercive policies (such as “vaccine passports”) led the un-vaccinated to become more convinced that jabs were a form of control, to become more *dis*trustful of authority^9^ and therefore to become more resistant to vaccination.^10^

 A combination of such micro-processes helps explain the macro-social findings that COVID-19 infection and fatality rates in different countries were more associated with levels of trust than with health service preparedness^11^ and that, while democracy was important to effective country responses, this was especially true where democracy was accompanied by trust.^12^ It follows that preparedness policies for future pandemics should pay as much attention to building trust (both before and during a crisis) as to boosting health service capacity, vaccine development and infection data gathering. So, when Mckee and colleagues declare “For too long, too many of those involved in health policy, as practitioners or researchers, have overlooked or undervalued the importance of trust. This must change”^13^ I can only applaud. Their contribution is a powerful call to arms.

 I equally agree when McKee et al conclude that: “commitments to respect and value trust and its core role in health system functioning and transformation that are not followed by sincere and meaningful actions will only fuel mistrust and undermine the trustworthiness of health systems.” Certainly, there is little value in recognising the importance of trust without acting to change levels of trust. In order to do this, it is necessary to understand how trust is generated and identify the barriers to change. My argument is premised on the assumption that however much policy-makers and practitioners recognise the importance of trust, actually doing so depends on understanding the principles and the pragmatics of trust-building.

## Trust and Shared Identity

 Trust is a notoriously complex construct to the extent that some have given up on even trying to define it.^14^ Here, we are concerned more specifically in trustworthiness.^15^ That is, our focus is the trust that one actor has in another actor. Accordingly, I define trust to mean an assumption that the other is able and motivated to act for the furtherance of my own self-interest. I draw in particular on the social identity approach, arguably the most influential contemporary approach to group processes,^16^ in order to argue that trust is best achieved when we extend our sense of self such that it includes both the subject and the other. When we think in terms of “we” rather than “I,” such that both myself and the other become included within the broader identity, then the interest of the other becomes my own and vice-versa. In other words, trust is intimately bound up with the creation of a sense of shared identity.^17^

 There is much evidence to support this claim.^18^ We pay more heed to those we regard as ingroup members, we respect them more, support them more and cooperate with them more.^19^ We even tolerate their criticisms more. That is because we trust that they are motivated to advance our shared interests even if we think that the way they are going about it is wrong. By contrast, we distrust the challenges of outgroup members as seeking to undermine the group interest.^20^

 It follows that the question of building trust resolves, at least in part, to the question of building shared identity. This has three dimensions: rhetorical, procedural and practical. The rhetorical involves the authorities talking in inclusive terms, whereby they and the public are referred to as part of a single category.^21^ At it is simplest it involved the use of “we” rather than “you” and “I.”^22^ But it also took many more nuanced forms and those leaders who spoke in terms of partnership, of interdependence and of shared responsibilities tended to fare better during the pandemic.^23^

 The performative involves the authorities acting in ways that signal that they see themselves and the public as equal partners in a common enterprise. At its simplest it involves policy-makers abiding by the rules they introduce for the public. Undoubtedly, the greatest loss of trust in the UK Government came when the Prime Minister, Boris Johnson endorsed his chief advisor, Dominic Cummings after the latter violated COVID-19 travel regulations. This fed a sense of “one law for us and another for them” amongst the public and was fatal for shared identity.^24^ But again, this was but one of many performative mis-steps, whereby Government acted in ways that communicated their distance from (rather than their commonality with the public). They failed to heed the principles of “procedural justice: treat people with respect, show concern for them, listen to them, heed their concerns.”^25^

 The final dimension of identity building involves the implementation of policies which bring people together in practice. Indeed, it is a foundational principle of social identity research that the psychological categories which organise our sense of “us” and “them” reflect the ways in which people are organised in the real world.^26^ As long as the public is divided by COVID-19 measures (some being able to abide by these measures and some not) then the result will be division, suspicion and conflict rather than unity, trust and solidarity.^27^

 This is all very well, but what does it mean in practice? What should authorities actually do in order to build trust? Here, I suggest four guidelines rooted in some of the major ways in which trust-building proved problematic during the pandemic.^28^

## Four Guidelines of Trust-Building

 Given my limited space, here I can only briefly sketch the guidelines. For more details along with supportive evidence to illustrate and justify what needs to be done, see Supplementary file 1.

###  Guideline 1: Trust the People!

 Trust is reciprocal. The public will not trust authority if authority talks and acts in ways that suggest that they do not trust the public and that the public is a burden rather than a partner in dealing with the crisis. Trust-building therefore requires discarding approaches to human psychology which suggest that people are Inherently flawed in their thinking, in their morality and in their resilience – and that all these flaws are exacerbated in an emergency. Particularly in an emergency, you need to trust the people!

###  Guideline 2: Recognise and Respect Difference

 Working with people means recognising the circumstances in which they act and which constrain what they are able to do. But different groups in the community face different circumstances. Hence building shared identity (and trust) does not mean treating everyone the same. It means recognising the different barriers to action that they face. It means treating people appropriately and tailoring interventions to their specific situation.

###  Guideline 3: Engage With the Public

 Identity and trust are rooted in doing things *with* people, not just doing things *for* people. Such engagement works in multiple ways. First, in itself, such partnership signifies respect and trust in people. Second, it is only through listening and learning that one can understand the barriers people face in adhering to crisis measures and address them accordingly. Third, by working through community leaders who are already trusted, what authority proposes is more likely to be trusted.

###  Guideline 4: Understanding and Support Trump Blame and Punishment

 How does one deal with non-adherence to measures implemented by authority. Blaming people for violations and imposing punishments may incentivise people to comply, but at the cost of demotivating them. Blame connotes lack of respect, lack of trust and ignorance of the barriers to compliance. Punishment is the ultimate failure of partnership. By contrast, understanding and support not only makes it possible to overcome the practical barriers to compliance but also communicates understanding and concern hence building identity and trust.

## Conclusion

 McKee and colleagues call for trust in public health.^13^ The principles and guidelines that I have outlined here, are intended to help us execute that call – or, in the language often used during the pandemic (and largely forgotten since) they outline how we can “build back better.”

 At the same time, my arguments point to a potential danger in our discussions of the issue. At worst, if one frames the (mis)trust issue as yet another way in which the public pose problems for crisis management, it can actually undermine the crisis response. As I have stressed throughout, the issue of trust must be nested within a recognition that the public are not part of the problem but part of the solution to pandemics and other such challenges, that this potential can only be realised by recruiting the public as a partner and that this means not just doing things for people but doing things with them. These are massive challenges. They require further paradigm changes in theory and in practice. They also require extending the interdisciplinary axis between medical, life and behavioural sciences so that it becomes the norm rather than an exceptional product of exceptional times.

## Ethical issues

 Not applicable.

## Conflicts of interest

 Author declares that he has no conflicts of interest.

## Supplementary files


Supplementary file 1. Four Guidelines for Trust-Building: Extended Outline.

## References

[1] Jetten J, Reicher SD, Haslam SA, Cruwys T. Together Apart: The Psychology of COVID-19. London: SAGE Publications; 2020.

[2] The Independent. Government Delayed Lockdown Over Fears of ‘Behavioural Fatigue’ – But Their Own Scientists Don’t Agree it Exists. https://www.independent.co.uk/news/uk/home-news/coronavirus-behavioural-fatigue-uk-lockdown-delay-science-chris-witty-robert-west-a9644971.html.

[3] Arnold KF, Gilthorpe MS, Alwan NA (2022). Estimating the effects of lockdown timing on COVID-19 cases and deaths in England: a counterfactual modelling study. PLoS One.

[4] Duffy B, Allington D. The Accepting, the Suffering, and the Resisting: The Different Reactions to Life Under Lockdown. The Policy Institute, Kings College London; 2020. https://www.kcl.ac.uk/policy-institute/assets/Coronavirus-in-the-UK-cluster-analysis.pdf.

[5] Wright L, Steptoe A, Fancourt D (2021). Predictors of self-reported adherence to COVID-19 guidelines A longitudinal observational study of 51,600 UK adults. Lancet Reg Health Eur.

[6] Atchison CJ, Bowman L, Vrinten C, et al. Perceptions and behavioural responses of the general public during the COVID-19 pandemic: a cross-sectional survey of UK Adults. medRxiv [Preprint]. April 3, 2020. https://www.medrxiv.org/content/10.1101/2020.04.01.20050039v1.full. 10.1136/bmjopen-2020-043577 PMC7783373 33397669

[7] Falkenbach M, Willison C (2022). Resources or trust: what matters more in the vaccination strategies of high-income liberal democracies?. Health Policy Technol.

[8] BBC. COVID: Turning Down COVID Vaccine is Selfish, Says Michael Gove. 2021. https://www.bbc.com/news/uk-politics-57987016.

[9] Jørgensen F, Bor A, Petersen MB (2024). Increased pressure lowered trust among unvaccinated during the COVID-19 pandemic: effects of the announcement of reintroducing vaccination passports in Denmark. Eur J Polit Res.

[10] de Figueiredo A, Larson HJ, Reicher SD (2021). The potential impact of vaccine passports on inclination to accept COVID-19 vaccinations in the United Kingdom: evidence from a large cross-sectional survey and modeling study. EClinicalMedicine.

[11] Bollyky TJ, Hulland EN, Barber RM (2022). Pandemic preparedness and COVID-19: an exploratory analysis of infection and fatality rates, and contextual factors associated with preparedness in 177 countries, from Jan 1, 2020, to Sept 30, 2021. Lancet.

[12] Bollyky TJ, Angelino O, Wigley S, Dieleman JL (2022). Trust made the difference for democracies in COVID-19. Lancet.

[13] McKee M, Schalkwyk MCV, Greenley R, Permanand G (2024). Placing trust at the heart of health policy and systems. Int J Health Policy Manag.

[14] Simpson TW (2012). What is trust?. Pac Philos Q.

[15] Robbins BG (2016). What is trust? A multidisciplinary review, critique, and synthesis. Sociol Compass.

[16] Haslam SA, Ellemers N, Reicher SD, Reynolds K, Schmitt MT. The social identity perspective tomorrow: opportunities and avenues for advance. In: Postmes T, Branscomne NR, eds. Rediscovering Social Identity: Core Issues. London: Routledge; 2010:357-380.

[17] Tanis M, Postmes T (2005). A social identity approach to trust: interpersonal perception, group membership and trusting behaviour. Eur J Soc Psychol.

[18] Tyler TR. Why do people rely on others? Social identity and social aspects of trust. In: Cook KS, ed. Trust in Society. Russell Sage Foundation. 2001:285-306.

[19] Reicher S, Spears R, Haslam SA (2010). The social identity approach in social psychology.

[20] Hornsey MJ (2005). Why being right is not enough: predicting defensiveness in the face of group criticism. Eur Rev Soc Psychol.

[21] Haslam SA, Reicher S, Platow MJ. The New Psychology of Leadership: Identity, Influence and Power. London: Routledge; 2020.

[22] Steffens NK, Haslam SA (2013). Power through ‘us’: leaders’ use of we-referencing language predicts election victory. PLoS One.

[23] Haslam SA, Steffens NK, Reicher SD, Bentley SV (2021). Identity leadership in a crisis: a 5R framework for learning from responses to COVID-19. Soc Issues Policy Rev.

[24] Fancourt D, Steptoe A, Wright L (2020). The Cummings effect: politics, trust, and behaviours during the COVID-19 pandemic. Lancet.

[25] Tyler TR, Blader SL (2003). The group engagement model: procedural justice, social identity, and cooperative behavior. Pers Soc Psychol Rev.

[26] Turner JC, Hogg MA, Oakes PJ, Reicher SD, Wetherell M. Rediscovering the Social Group: A Self-Categorization Theory. Oxford: Basil Blackwell; 1987.

[27] Jetten J, Reicher SD, Haslam SA, Cruwys T. Together Apart: The Psychology of COVID-19. London: SAGE Publications; 2020.

[28] Reicher S, Bauld L (2021). From the ‘fragile rationalist’ to ‘collective resilience’: what human psychology has taught us about the COVID-19 pandemic and what the COVID-19 pandemic has taught us about human psychology. J R Coll Physicians Edinb.

