# Supplementary material for: Principles and Pragmatics for Building Trust in Authority: Comment on "Placing Trust at the Heart of Health Policy and Systems"
Source: Int J Health Policy Manag. 2025 Apr 23;14:8964. doi: 10.34172/ijhpm.8964 (PMC12257189; doi:10.34172/ijhpm.8964)
Supplement: Supplementary file 1 — Four Guidelines for Trust-Building: Extended Outline. [file ijhpm-14-8964-s001.pdf]

Article title: Principles and pragmatics for building trust in authority
Journal name: International Journal of Health Policy and Management (IJHPM)
Authors' information: Stephen David Reicher*
School of Psychology and Neuroscience, University of St. Andrews, St. Andrews, UK.
***Correspondence to:** Stephen David Reicher; Email: sdr@st-andrews.ac.uk
Citation: Reicher SD. Principles and pragmatics for building trust in authority: Comment on "Placing trust at the heart of health policy and systems." Int J Health Policy Manag. 2025;14:8964. doi:10.34172/ijhpm.8964
Supplementary file 1. Four Guidelines for Trust-Building: Extended Outline

Guideline 1: Trust the people!

In his Proverbs for Paranoids, Thomas Pynchon notes that "if they can get you asking the wrong questions, they don't need to worry about the answers"[1]. When it comes to trust, the concern of authorities is characteristically "why do they (the public) mistrust us". This leads to problems in how authorities talk about (and to) the public and also to problems in the way they do (and don't) treat the public.

All these problems stem from the fact that trust is reciprocal[2]. The public won't trust authorities unless authorities trust the public, the latter question receiving relatively scant attention. As a consequence, public distrust is characteristically invoked with a sense of exasperation, as if their wariness is unreasonable, denotes a problem in *them* and makes them a problem for us. Yet if there is mistrust, there is often good reason for it. For instance, mistrust of medical interventions amongst African-Americans is rooted in a long historical experience of abuse[3]. Certain notorious examples (such as the Tuskagee syphilis experiment in which black men were deliberately withheld medication for the disease over a period of 40 years[4]) are widely known in the Black community and those members who have greater knowledge of this past are more distrustful of present interventions[5].

All in all, framing the trust issue as public mistrust of authority not merely ignores the key issue of how authorities mistrust the public but also communicates such mistrust to the public. It tells members of the public that the authorities regard them as irrational and unreasonable. It denies their experiences of authority. It therefore contributes to creating mistrust of authority in the public.

As for why authorities mistrust the public, there are many answers on many levels. One is a set of paternalistic assumptions according to which the public do not know their own minds and are unable to assess their own interests, particularly in a crisis, and therefore need enlightened authorities to direct them. Such paternalism, even if overtly repudiated, remains widespread in political and also in medical circles[6]. It's assumptions also underpin models of human behaviour which have been very influential, especially amongst government, in recent years[7].

Notably, the notion of 'nudge' derives from the perception that human thinking is inherently flawed and biased"[8]. As a consequence, all one can do to shape behaviour is leverage these flaws (such as our tendency to do what is easy rather than what is best for us).[9] An example of a nudge might be to try and encourage healthy eating by simple interventions such as putting fruit by the check-out desk.

The danger of such approaches is less to do with the practices they rule in as in those they rule out. These approaches convey that there is no point in engaging with people and reasoning with them because they are incapable of good reasoning. There is no point in providing information about what is good for them because people can't understand what is good for them. The public can never be a partner and part of the solution to pandemic challenges. They are, by their very nature, part of the problem that the authorities have to manage. Where such perceptions inform policy and practice – from the failure to engage with marginalised communities on vaccine take up to the failure to provide adequate information about the possible side-effects of vaccines – the message (both rhetorical and performative) to the public is loud and clear: 'we don't trust you'. At which point, why would the public respond 'but we do trust you'?

Guideline 2:: Recognise and respect difference

Trusting the public may be a precondition for the authorities to partner with the public and, through treating the pandemic response as a joint enterprise, generate shared identity and trust (which in turn reinforces effective co-action – a virtuous cycle). However it is not sufficient. There are a number of further conditions and these underpin both this and the next two guideliness.

With the best will in the world, you cannot work with people unless you take account of their circumstances and the way that any given measure will impact differentially on those in different circumstances. This leads to a seemingly paradoxical conclusion. Although the aim of building shared identity will all sections of the community is to do with overcoming difference and creating

a sense of ‘we are all in this together’, this cannot be achieved by treating everyone in the same way. To the contrary, it requires that they are treated differently.

Early on during the pandemic, Bonnie Henry – Chief Medical Officer in British Columbia – famously noted that we may all be in the same storm, but we are not all in the same boat[10]. On the level of personal experience, COVID was not a single event but created a series of different realities for different groups. For instance, stay-at-home orders were experienced very differently by those with large houses and gardens (many of whom recalled the ‘lockdown’ with nostalgia[11]) compared to those living in high-rise blocks with small children and no outdoor space. The differences equally impacted health and mortality. For instance, at the start of the pandemic, black men were dying at three times the rate of white men[12]. In this context, for well-heeled politicians to insist ‘we are all in this together’ merely highlighted how unaware they were of the realities of life for many, accentuated the sense of difference and distance between authority and these publics, rendered their interventions ineffective (for some at least), corroded shared identity and undermined trust.

The same issues apply to the introduction of COVID laws, rules and regulations in general. If people are required or asked to do something that they have difficulty doing, then not only are they less likely to do it, but also the requirement signals and exacerbates their alienation from authority. If people are told to get vaccinated when it is difficult for them to access vaccination centres, if they are told the vaccine might make them feel poorly for a day or two when they can’t afford to take time off work, if they are told to self-isolate at home when they live in crowded accommodation sharing bedrooms and bathrooms, then all these demands simply signal that those doing the demanding don’t understand their problems and/or do not care about them. In yet another way, It says that authority is neither of them nor for them.

This applies not only at an individual level but at a collective level. For instance, when the UK introduced a rule to say that no more than six people could meet together outdoors it discriminated against larger families who then could not meet with others without breaching the limit[13]. It thereby discriminated specifically against certain ethnic and racial minorities, such as Black people, who generally live in larger family groups[14]. This, is one of many factors (such as the historical legacies outlined under guideline 1) which help explain why the black population had lower trust in authority during COVID and had lower confidence that the COVID response was designed with the needs of Black people in mind[15].

In order to build shared identity and trust, therefore, the key thing is to ensure everyone is equally able to do what is asked of them. This means understanding and respecting the circumstances in which people act and tailoring interventions to these circumstances. It means treating people appropriately, not all the same.

In practical terms, if you want to build trust with the entire community, then make sure, in planning your interventions, that you consider how they impact different groups, what are the barriers to adherence amongst these groups, and how can group members can be helped to overcome these barriers.

Guideline 3: Engage with the public

If one wants to understand and address the requirements of different groups in the community (and thereby avoid inadvertently alienating them), then it is essential to listen to them. But listening is not only a means of crafting interventions that maintain trust. It also has a direct impact on trust. Listening is a central element of procedural justice[16] and being seen to have an interest in what others say is a critical element in creating a sense of ingroupness[17]. Authorities that listen are more likely to be listened to, still more if they act on what they hear[18]. During the pandemic, for instance, initiatives of engagement which went through community organisations, which gave people space to voice their concerns, and which answered those concerns, were highly effective in increasing vaccination rates, especially amongst communities that were more distrustful of authority[19].

However, it is important to avoid too simple and too naïve an association between listening, engagement and trust[20]. In particular, and especially when engagement initiatives are limited to consultation, they can be devices that facilitate authorities in deciding how best to impose their own agenda on the public. At worst, such consultation exercises can be a means of avoiding debate and accountability. They allow those in charge to solicit a plethora of views from which they then pick and choose those which accord with what they favoured in advance[21].

To put it slightly differently, consultation must be distinguished from empowerment[22]. To be genuinely empowering, engagement activities must enable the public to define and advance their own agendas – not only reacting to what the authorities propose but defining the problems that concern them and offering solutions. Hence, the earlier in the policy process that engagement occurs and the more the terms of engagement are mutually constituted – in other words, the more it is a genuine partnership – then the more likely it is to succeed in building positive social relations between authorities and the public.

Guideline 4: Understanding and support trump blame and punishment.

Let us assume that, as an authority, you recognise the importance of building a sense of shared identity in order to generate trust), that in order to do so you trust the public (guideline 1), recognise their (different) circumstances (guideline 2) and listen to them in order to understand the impact of those circumstances (guideline 3). Still, all of that will be of little use if you don't then provide the practical support which allows them to meet the demands placed upon them. Moreover, the importance of support lies not only in enabling people to adhere to requirements but also motivating them to do so by showing that authorities both understand and are concerned for them. That is, to repeat my basic mantra of trust building, it shows that authorities are of the people and for the people.

To put it more briefly, what I am advocating is an approach to the public based on understanding and support rather than one rooted in blame and punishment. This contrast can be illustrated by the very first response to the pandemic in the UK. Within a week of the COVID stay-at-home regulations being introduced in March 2020, a new word entered the lexicon: Covidiots. Newspapers carried photos of crowds in public spaces – such as commons and parks – as evidence of public stupidity[23] and calls were made for people to be barred from such spaces[24]. From the start, then, the dominant response was one of blame and punishment. This only became more entrenched with time. In September 2020 as infections began to rise again, Prime Minister Boris Johnson blamed the increase on people "brazenly defying" the rules and imposed fines of up to £10,000 for doing so[25]. The problem with such approaches is that they do little to secure compliance. Worse, they position the public as driven by ill-will, as a problem and as 'other' to authority.

What is more, blame and punishment attribute (mis)behaviour to motivation when, as we have seen, violations of COVID regulations often had more to do with resource constraints. In the case of crowding in parks, people had been told they could go outside for their health. The problem, especially in urban settings, is that there are limited public spaces where they could go. So, inadvertently, people found themselves crowded together. To limit those spaces still further by closing the parks merely compounded the issue. A better solution would have been to make more spaces (e.g. school playing fields) available[26].

Understanding people's situation and supporting them to do the right thing enables compliance. Moreover, showing an understanding of the difficulties faced by a people and a commitment to

mitigating those difficulties improves rather than corrodes relations between the public and authority. For instance, we have (as yet) unpublished experimental data to show that offering support for self-isolation increases levels of intended self-isolation both by making it viable and by making people think that Government is on their side.

So why, then, despite repeated calls for the Government to increase support for self-isolation did it not happen? The answer, given by Health Secretary Matt Hancock to a Parliamentary Inquiry, was a fear that people would 'game the system[27]'. It would be hard to find a more depressing or a more egregious example of the distrust with which Government view the public, the way it underpins practices (such as blame and punishment) that undermine the trust of the public in Government and the way it impedes trust-building practices (such as making it practically possible to obey covid regulations).

---

[1] Pynchon, T. (1973) *Gravity's Rainbow*. New York: Viking.

[2] Serva, M. A., Fuller, M. A., & Mayer, R. C. (2005). The reciprocal nature of trust: A longitudinal study of interacting teams. *Journal of Organizational Behavior: The International Journal of Industrial, Occupational and Organizational Psychology and Behavior*, *26*(6), 625-648.

[3] Dula, A. (1994). African American suspicion of the healthcare system is justified: what do we do about it? Cambridge quarterly of healthcare ethics, 3(3), 347-357.

[4] Gray, F.D. (1998). *The Tuskegee Syphilis Study: The Real Story and Beyond*. Montgomery, Alabama: NewSouth Books.

[5] Nelson, J. C., Adams, G., Branscombe, N. R., & Schmitt, M. T. (2010). The role of historical knowledge in perception of race-based conspiracies. *Race and Social Problems*, *2*, 69-80.

[6] Lynøe, N., Engström, I. & Juth, N. (2021) How to reveal disguised paternalism: version 2.0. *BMC Medical Ethics 22*, 170.

[7] OECD (2017) Behavioural insights and public policy. https://www.oecd.org/en/publications/behavioural-insights-and-public-policy_9789264270480-en.html See also Sodha, S. (2020) Nudge theory is a poor substitute for hard science in matters of life and death. The Guardian, 26th April. https://www.theguardian.com/commentisfree/2020/apr/26/nudge-theory-is-a-poor-substitute-for-science-in-matters-of-life-or-death-coronavirus

[8] Thaler, R. H. (1991) *Quasi-Rational Economics*. Russell Sage Foundation, 1991. p.4.

[9] Sunstein, C. R. (2022) The distributional effects of nudges. *Nature Human Behaviour 6,* 9–10.

[10] https://vancouversun.com/opinion/op-ed/dr-bonnie-henry-as-we-venture-out-into-the-world-again-lets-do-so-steadily-but-cautiously

[11] See, for instance https://www.dailymail.co.uk/news/article-8461895/Middle-class-parents-ENJOYED-lockdown-study-finds.html and https://www.ft.com/content/6abb2556-3f33-4425-bc52-2c02a0e7f32d

[12] https://www.ons.gov.uk/peoplepopulationandcommunity/birthsdeathsandmarriages/deaths/articles/coronavirusCOVID19relateddeathsbyethnicgroupenglandandwales/2march2020to15may2020

[13] https://www.bbc.co.uk/news/uk-england-54101524

[14] Race Equality Foundation (2020) Coronavirus (COVID-19) and how it affects Black, Asian and minority ethnic people and communities. https://raceequalityfoundation.org.uk/health-and-care/lockdown-2-briefing/

[15] Rusoja, E. A., & Thomas, B. A. (2021). The COVID-19 pandemic, Black mistrust, and a path forward. EClinicalMedicine, 35.

[16] Lind, E. & Tyler, T. (1988). *The Social Psychology of Procedural Justice*. New York: Plenum Press.

[17] Lee, G. C., Platow, M. J., & Cruwys, T. (2024). Listening quality leads to greater working alliance and well-being: Testing a social identity model of working alliance. *British Journal of Clinical Psychology*.

[18] Burgess, R. A., Osborne, R. H., Yongabi, K. A., Greenhalgh, T., Gurdasani, D., Kang, G., ... & McKee, M. (2021). The COVID-19 vaccines rush: participatory community engagement matters more than ever. *The Lancet*, *397*(10268), 8-10.

[19] https://www.ovg.ox.ac.uk/news/COVID-19-how-to-tackle-vaccine-hesitancy-among-bame-groups

[20] Petts, J. (2008). Public engagement to build trust: false hopes?. *Journal of Risk Research*, *11(6)*, 821-835.

[21] Arnstein, S. R. 1969. A ladder of citizen participation. *Journal of the American Institute of Planners, 35***,** 216-224.

[22] Hammond, M. 2020. Democratic innovations after the post-democratic turn: between activation and empowerment. *Critical Policy Studies***,** 15(2), 174-191

[23] https://www.dailymail.co.uk/news/article-8138439/Public-urged-away-UK-holiday-destinations-limit-COVID-19-spread.html

[24] https://www.telegraph.co.uk/news/2021/03/31/uk-weather-dont-blow-matt-hancock-warns-temperatures-near-march/

[25] https://www.theguardian.com/commentisfree/2020/sep/25/johnson-sturgeon-public-compliance

[26] https://www.theguardian.com/world/2020/apr/12/private-schools-land-targeted-for-families-without-gardens

[27] https://www.independent.co.uk/news/uk/politics/self-isolation-support-matt-hancock-b1863369.html
